# Complete genome sequence of *Leuconostoc suionicum* DSM 20241^T^ provides insights into its functional and metabolic features

**DOI:** 10.1186/s40793-017-0256-0

**Published:** 2017-07-17

**Authors:** Byung Hee Chun, Se Hee Lee, Hye Hee Jeon, Dong-Woon Kim, Che Ok Jeon

**Affiliations:** 1 Department of Life Science, Chung-Ang University, 84, HeukSeok-Ro, Dongjak-Gu, Seoul, 06974 Republic of Korea; 2 Microbiology and Functionality Research Group, World Institute of Kimchi, Gwangju, 61755 Republic of Korea; 3 Animal Nutrition and Physiology Team, National Institute of Animal Science, RDA, Jeollabukdo, 55365 Republic of Korea

**Keywords:** *Leuconostoc suionicum*, Complete genome, Lactic acid bacteria, KEGG, Fermentative metabolic pathway

## Abstract

The genome of *Leuconostoc suionicum* DSM 20241^T^ (=ATCC 9135^T^ = LMG 8159^T^ = NCIMB 6992^T^) was completely sequenced and its fermentative metabolic pathways were reconstructed to investigate the fermentative properties and metabolites of strain DSM 20241^T^ during fermentation. The genome of *L. suionicum* DSM 20241^T^ consists of a circular chromosome (2026.8 Kb) and a circular plasmid (21.9 Kb) with 37.58% G + C content, encoding 997 proteins, 12 rRNAs, and 72 tRNAs. Analysis of the metabolic pathways of *L. suionicum* DSM 20241^T^ revealed that strain DSM 20241^T^ performs heterolactic acid fermentation and can metabolize diverse organic compounds including glucose, fructose, galactose, cellobiose, mannose, sucrose, trehalose, arbutin, salcin, xylose, arabinose and ribose.

**Electronic supplementary material:**

The online version of this article (doi:10.1186/s40793-017-0256-0) contains supplementary material, which is available to authorized users.

## Introduction

The genus *Leuconostoc* comprises Gram-positive, facultatively anaerobic, intrinsically vancomycin-resistant, catalase-negative, spherical heterofermentative lactic acid bacteria which are involved in the fermentation of plant materials (such as kimchi), dairy products, meats, vegetable sausages and beverages [1–7]. Strain DSM 20241 ^T^ (=ATCC 9135 ^T^ =LMG 8159 ^T^ =NCIMB 6992 ^T^) of the genus *Leuconostoc* was isolated in Sweden in 1972. It was originally classified as a subspecies of *L. mesenteroides*, but was recently reclassified as a novel species –*L. suionicum*–based on its whole genome sequence [4]. Here, we present the taxonomic and genomic features of *L. suionicum* DSM 20241 ^T^. In addition, we investigated the metabolic properties of *L. suionicum* DSM 20241 ^T^ and reconstructed the metabolic pathways of organic compounds to estimate the fermentative metabolites in *L. suionicum* DSM 20241 ^T^.

## Organism information

### Classification and features

**Fig. 1:**
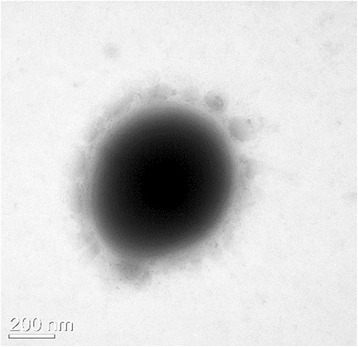
Transmission electron micrograph showing the general cell morphology of Leuconostoc suionicum DSM 20241^T^. The bacterial cells were stained by uranyl acetate and examined using transmission electron microscopy (JEM-1010; JEOL)

*L. suionicum* DSM 20241 ^T^ belongs to the family *Leuconostocaceae*, order *Lactobacillales*, class *Bacilli* and phylum *Firmicutes*. Strain DSM 20241 ^T^ is a Gram-positive, facultatively anaerobic, non-motile, non-sporulating, catalase-negative coccus, with a diameter of 0.5–0.7 μm (Fig. 1). It can be grown in MRS broth at 10–40 °C, with an optimal growth temperature of 30 °C [4]. Strain DSM 20241 ^T^ ferments a wide variety of carbon sources including d-glucose, arbutin, melibiose, sucrose, turanose, *N*-acetylglucosamine, cellobiose, galactose, gentiobiose, amygdalin, l-arabinose, esculin, ferric citrate, d-fructose, d-mannose, lactose, maltose, methyl *α*-d-glucopyranoside, salicin, trehalose, d-xylose, potassium 5-ketogluconate, mannitol and ribose to produce gas and acids (Table 1); however, it does not ferment glycerol, erythritol, d-arabinose, l-xylose, d-adonitol, methyl *β*-d-xylopyranoside, l-sorbose, methyl *α*-d-mannopyranoside, l-rhamnose, dulcitol, inositol, d-sorbitol, inulin, d-melezitose, starch, glycogen, xylitol, d-lyxose, d-tagatose, fucose, d-arabitol, l-arabitol, potassium gluconate, potassium 2-ketogluconate or raffinose [4, 8].

**Table 1:** Classification and general features of *Leuconostoc suionicum* DSM 20241^T^ according to MIGS recommendations [9]

MIGS ID	Property	Term	Evidence code^a^
	Classification	Domain *Bacteria*	TAS [31]
		Phylum *Firmicutes*	TAS [32, 33]
		Class *Bacilli*	TAS [34]
		Order *Lactobacillales*	TAS [35]
		Family *Leuconostocaceae*	TAS [35]
		Genus *Leuconostoc*	TAS [36–38]
		Species *Leuconostoc suionicum*	TAS [4]
		Type strain DSM 20241^T^	TAS [4]
	Gram stain	Positive	TAS [8]
	Cell shape	Coccus	TAS [8]
	Motility	Non-motile	NAS
	Sporulation	Non-sporulating	TAS [8]
	Temperature range	10–40 °C	TAS [4]
	Optimum temperature	30 °C	TAS [4, 8]
	pH range; Optimum	Not reported	
	Carbon source	l-arabinose, ribose, d-xylose, galactose, glucose, fructose, mannose, methyl α-d-glucopyranoside, *N*-acetylglucosamine, amygdalin, arbutin, aesculin, salicin, cellobiose, maltose, melibiose, sucrose, trehalose, gentiobiose and turanose	TAS [4, 8]
MIGS-6	Habitat	Not reported	
MIGS-6.3	Salinity	Not reported	
MIGS-22	Oxygen requirement	Facultatively anaerobic	TAS [8]
MIGS-15	Biotic relationship	Free-living	NAS
MIGS-14	Pathogenicity	Not reported	NAS
MIGS-4	Geographic location	Sweden	TAS [8]
MIGS-5	Sample collection	1972	TAS [8]
MIGS-4.1	Latitude	Not reported	
MIGS-4.2	Longitude	Not reported	
MIGS-4.4	Altitude	Not reported	

^a^Evidence codes - *IDA* Inferred from Direct Assay, *TAS* Traceable Author Statement (i.e., a direct report exists in the literature), *NAS* Non-traceable Author Statement (i.e., not directly observed for the living, isolated sample, but based on a generally accepted property for the species, or anecdotal evidence). These evidence codes are from the Gene Ontology project [cite this reference]

**Fig. 2:**
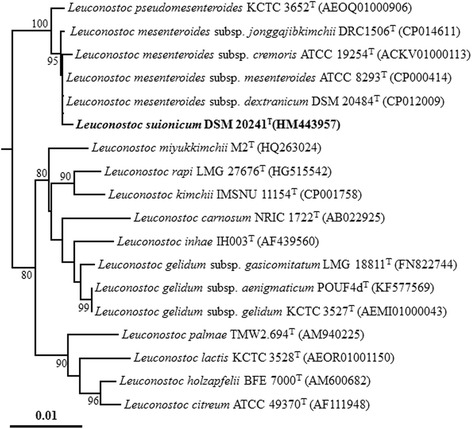
Neighbor-joining tree based on the 16S rRNA gene sequences showing the phylogenetic relationships between Leuconostoc suionicum DSM 20241^T^ (*highlighted in bold*) and closely related Leuconostoc species. The sequences were aligned using the fast secondary-structure aware Infernal aligner available from the Ribosomal Database Project [28] and the tree was constructed based on the neighbor-joining algorithm using PHYLIP software (ver. 3.68) [29]. Bootstrap values of over 70% are shown on the nodes as percentages of 1000 replicates. Weissella viridescens 1536^T^ (AB023236) was used as an outgroup (not shown). *Bar* indicates 0.01 changes per nucleotide position

Phylogenetic analysis using the 16S rRNA gene sequences with validated type strains showed that *L. suionicum* DSM 20241 ^T^ is most closely related to the subspecies of the species *L. mesenteroides*: *L. mesenteroides subsp. mesenteroides*, *L. mesenteroides subsp. jonggajibkimchii*, *L. mesenteroides subsp. cremoris*, and *L. mesenteroides subsp. dextranicum* with very high 16S rRNA gene sequence similarities (>99.73%; Fig. 2).

## Genome sequencing information

### Genome project history

*L. suionicum* DSM 20241 ^T^ was selected owing to its taxonomic significance for the species *L. mesenteroides* and was obtained from the German Collection of Microorganisms and Cell Cultures. The complete sequences of the chromosome and plasmid of strain DSM 20241 ^T^ were deposited in GenBank with the accession numbers CP015247–48. The project information and its association with MIGS version 2.0 [9] are summarized in Table 2.

**Table 2:** Genome sequencing project information for *Leuconostoc suionicum* DSM 20241^T^

MIGS ID	Property	Term
MIGS 31	Finishing quality	Complete
MIGS-28	Libraries used	PacBio 10-kb SMRT-bell library
MIGS 29	Sequencing platforms	PacBio RS SMRT
MIGS 31.2	Fold coverage	50 ×
MIGS 30	Assemblers	RS_HGAP Assembly.3
MIGS 32	Gene calling method	NCBI Prokaryotic Genome, Annotation Pipeline
	Locus Tag	A6B45
	GenBank ID	CP015247-CP015248
	GenBank Date of Release	14-APR-2017
	GOLD ID	Ga0151201
	BIOPROJECT	PRJNA318320
MIGS 13	Source Material Identifier	DSM 20241^T^/ ATCC 9135^T^/LMG 8159^T^/NCIMB 6992^T^
	Project relevance	Taxonomy, industry, fermentation

### Growth conditions and genomic DNA preparation

*L. suionicum* DSM 20241 ^T^ was cultured in MRS broth (BD Biosciences, CA, USA) at 30 °C for 24 h until the early stationary phase. Genomic DNA was extracted according to a standard phenol-chloroform extraction and ethanol precipitation procedure [10]. DNA quality (OD260/OD280 > 1.8) and concentration were measured using a NanoDrop ND-1000 spectrophotometer (Synergy Mx, Biotek, VT, USA).

### Genome sequencing and assembly

**Fig. 3:**
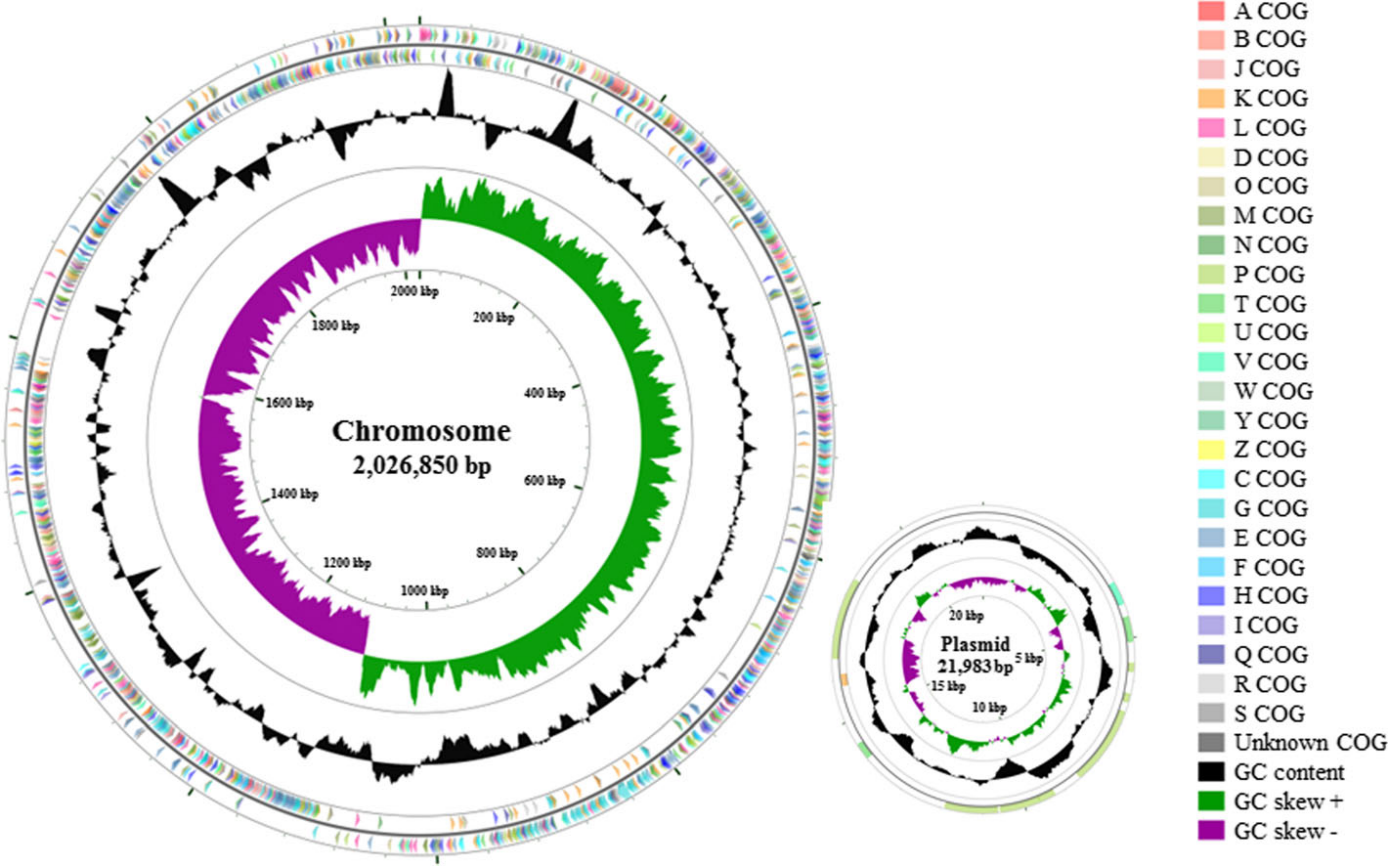
Graphical maps of the Leuconostoc suionicum DSM 20241^T^ chromosome and plasmid. The circular maps were set up by the CGView Server [30]. From the outside to the center: Genes on forward strand (colored by COG categories), genes on reverse strand (colored by COG categories), GC content (in *black*) and GC skews, where *green* indicates positive values and *magenta* indicates negative values

The genome of strain DSM 20241 ^T^ was sequenced using PacBio RS SMRT technology based on a 10-kb SMRT-bell library at Macrogen (Seoul, Korea) as previously described [10]; 138,738 high-quality reads were generated, with an average length of 7656 bp. De novo assembly of sequencing reads derived from PacBio SMRT sequencing was performed using the hierarchical genome assembly process (HGAP; ver. 3.0) [11], which yielded a circular chromosome (2,026,850 bp) and a circular plasmid (21,983 bp) (Fig. 3).

### Genome annotation

Automated genome annotation of strain DSM 20241 ^T^ was performed using Prodigal as part of the Joint Genome Institute’s microbial genome annotation pipeline [12]. In addition, predicted coding sequences were functionally annotated using the NCBI non-redundant database, UniProt, TIGR-Fam, Pfam, PRIAM, Kyoto Encyclopedia of Genes and Genomes, Clusters of Orthologous Groups, and InterPro. Structural RNA genes were identified by using HMMER 3.0rc1 (rRNAs) [13] and tRNAscan-SE 1.23 (tRNAs) [14]. Other non-coding genes were searched using INFERNAL 1.0.2 [15]. Additional annotation was performed within the Integrated Microbial Genomes—Expert Review platform [16].

## Genome properties

The complete genome of *L. suionicum* strain DSM 20241 ^T^ consists of a circular chromosome (2,026,850 bp) and a circular plasmid (21,983 bp) with 37.6% and 37.0% G + C contents, respectively (Table 3). The genome contains 1997 protein coding genes and 93 RNA genes (72 tRNAs, 12 rRNAs and 9 other RNAs; Table 4). Additional genome statistics and the distribution of the genes into COG functional categories are presented in Tables 4 and 5, respectively.

**Table 3:** Sequence features of chromosome and plasmid present in the *L. suionicum* DSM 20241^T^ genome

Label	Size (bp)	Topology	Coding gene sequences (bp)	G + C content (%)	INSDC identifier
Chromosome	2,026,850	Circular	1,758,165	37.6	CP015247.1
Plasmid	21,983	Circular	14,895	37.0	CP015248.1

**Table 4:** Genome statistics

Attribute	Value	% of Total
Genome size (bp)	2,048,833	100.00
DNA coding (bp)	1,835,796	89.60
DNA G + C (bp)	769,980	37.58
DNA scaffolds	2	100.00
Total genes	2090	100.00
Protein coding genes	1997	95.55
RNA genes	93	4.45
Pseudo genes	0	–
Genes in internal clusters	381	18.23
Genes with function prediction	1641	78.52
Genes assigned to COGs	1483	70.96
Genes with Pfam domains	1695	81.10
Genes with signal peptides	31	1.48
Genes with transmembrane helices	592	28.33
CRISPR repeats	0	–

**Table 5:** Number of genes associated with general COG functional categories

Code	Value	%age	Description
J	176	7.91	Translation, ribosomal structure and biogenesis
A	0	0.00	RNA processing and modification
K	116	5.21	Transcription
L	83	3.73	Replication, recombination and repair
B	0	0.00	Chromatin structure and dynamics
D	25	1.12	Cell cycle control, Cell division, chromosome partitioning
V	34	1.53	Defense mechanisms
T	53	2.38	Signal transduction mechanisms
M	93	4.18	Cell wall/membrane biogenesis
N	11	0.49	Cell motility
U	14	0.63	Intracellular trafficking and secretion
O	55	2.47	Posttranslational modification, protein turnover, chaperones
C	53	2.38	Energy production and conversion
G	146	6.56	Carbohydrate transport and metabolism
E	179	8.04	Amino acid transport and metabolism
F	85	3.82	Nucleotide transport and metabolism
H	98	4.40	Coenzyme transport and metabolism
I	65	2.92	Lipid transport and metabolism
P	81	3.64	Inorganic ion transport and metabolism
Q	27	1.21	Secondary metabolites biosynthesis, transport and catabolism
R	127	5.71	General function prediction only
S	98	4.40	Function unknown
-	607	27.27	Not in COGs

The total is based on the total number of protein coding genes in the genome

## Insights from the genome sequence

### KEGG metabolic and regulatory pathways

**Fig. 4:**
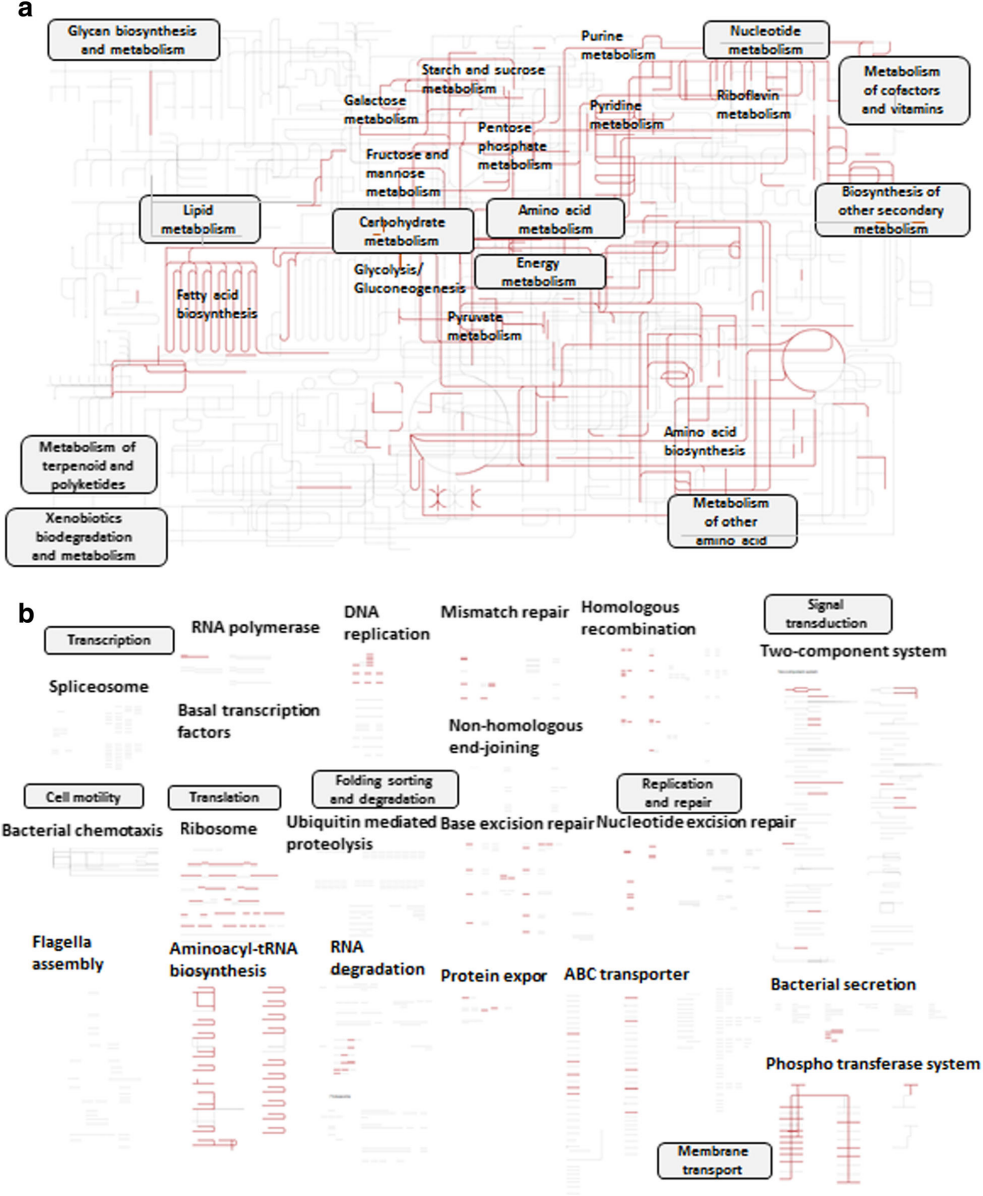
KEGG metabolic (**a**) and regulatory (**b**) pathways of *Leuconostoc suionicum* DSM 20241^T^ *.* The pathways were generated using the iPath v2 module based on KEGG Orthology numbers of genes identified from the genome of *L. suionicum* DSM 20241^T^

The KEGG metabolic pathways of *L. suionicum* DSM 20241 ^T^ show that strain DSM 20241 ^T^ displays typical heterolactic acid fermentative capabilities, performing pentose phosphate metabolism, fructose and mannose metabolism, galactose metabolism, sucrose metabolism and pyruvate metabolism without the complete tricarboxylic acid cycle (Fig. 4a, see Additional file 1: Table S1) [17–19]. In addition, *L. suionicum* DSM 20241 ^T^ harbors genes related to riboflavin metabolism, fatty acid biosynthesis, purine and pyrimidine metabolism and amino acid biosynthesis (Fig. 4a). The regulatory pathways of strain DSM 20241 ^T^ indicate that it contains various phospho transferase systems, such as a sucrose-specific EII component (K02808, K02809 and K02810), a *β*-glucoside *β*-glucoside-specific EII component (K02755, K02756 and K02757), a cellobiose-specific EII component (K02759, K02760 and K02761), a mannose-specific EII component (K02793, K02794, K02795 and K02796) and an l-ascorbate-specific EII component (K02821, K02822 and K03475) (Fig. 4b), suggesting that strain DSM 20241 ^T^ possesses the ability to ferment various carbon sources.

### Carbon metabolic pathways

**Fig. 5:**
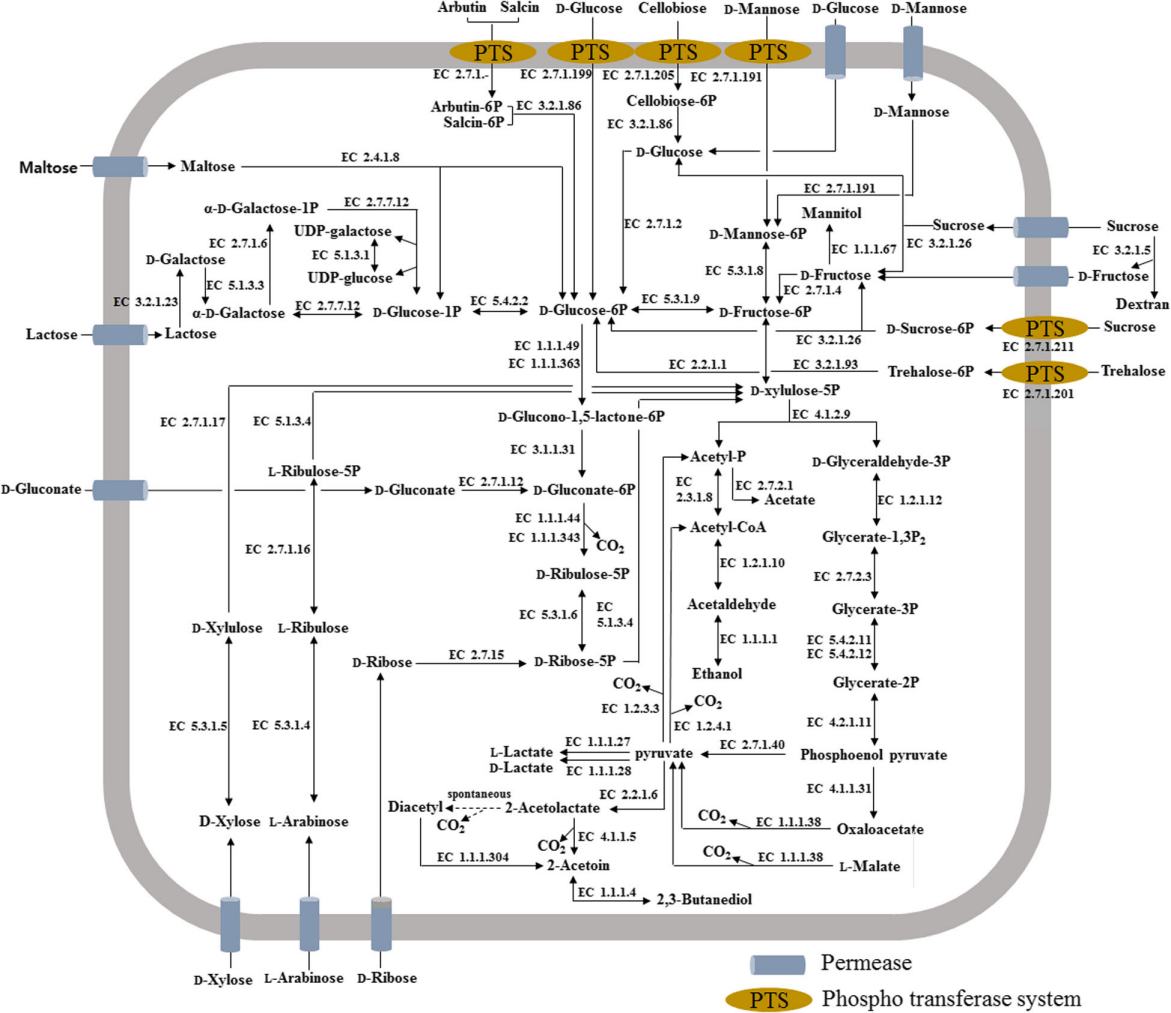
Predicted fermentative metabolic pathways of various carbon compounds in *Leuconostoc suionicum* DSM 20241^T^ during fermentation. PTS, phosphotransferase system; UDP, uridine diphosphate

To investigate the fermentative metabolic properties of *L. suionicum* DSM 20241 ^T^, metabolic pathways of various carbon sources were reconstructed based on predicted KEGG pathways and BLASTP analysis using reference protein sequences (Fig. 5). The predicted metabolic pathways identified motifs associated with the pentose phosphate pathway, fructose and mannose metabolism, galactose metabolism, sucrose metabolism, pyruvate metabolism, partial TCA cycle and incomplete glycolysis pathway in the genome of *L. suionicum* DSM20241 ^T^, indicating that this strain performs typical heterolactic acid fermentation to produce lactate, ethanol and carbon dioxide (Fig. 5, Additional file 1: Table S1). It has been reported that mannitol, an important refreshing sweet agent in fermented vegetable foods such as sauerkraut, pickles and kimchi, is synthesized through fructose reduction by mannitol dehydrogenase (EC 1.1.1.67) through the consumption of NADH [20, 21]. The predicted metabolic pathways indicate that *L. suionicum* DSM 20241 ^T^ produces ethanol via the reduction of acetyl phosphate through the consumption of NADH; this strain may also produce acetate instead of ethanol due to the lack of NADH when the strain produces mannitol from fructose [21]. *L. suionicum* DSM 20241 ^T^ harbors genes related to diverse PTSs or permeases that transport various glycosides or sugars including d-glucose, d-fructose, sucrose, d-mannose, trehalose, arbutin, salcin, cellobiose, d-xylose, arabinose, and d-ribose; this indicates that *L. suionicum* DSM 20241 ^T^ has versatile metabolic capabilities. d-lactate and l-lactate are produced from the reduction of pyruvate by d-lactate dehydrogenase (EC 1.1.1.28) and l-lactate dehydrogenase (EC 1.1.1.27), respectively*.* *L. suionicum* DSM 20241 ^T^ harbors four copies of d-lactate dehydrogenase (locus tags: Ga0151201_111849, Ga0151201_112070, Ga0151201_11385 and Ga0151201_111758) and one copy of l-lactate dehydrogenase (locus tag: Ga0151201_1175), suggesting that *L. suionicum* DSM 20241 ^T^ may produce more d-lactate than l-lactate; this is similar to other members of the genus *Leuconostoc*, which have been shown to produce more d-lactate than l-lactate under laboratory conditions [4, 22–25]. The predicted metabolic pathways show that *L. suionicum* DSM 20241 ^T^ produces diacetyl and acetoin, which are known as butter flavors in dairy products [26, 27]. Acetolactate synthase (EC 2.2.1.6) produces 2-acetolactate from pyruvate and converts it into deacetyl and CO_2_, which is emitted as a byproduct. Furthermore, 2-acetoin is produced from 2-acetolactate and diacetyl (acetolactate decarboxylase, EC 4.1.1.5; diacetyl reductase, EC 1.1.1.304, respectively); but 2-acetoin is eventually converted to 2,3-butanediol, which lacks the butter flavoring property. In addition, the predicted metabolic pathways show that *L. suionicum* DSM 20241 ^T^ uses dextransucrase (EC 2.4.1.5) to produce dextran, a homopolysaccharide of glucose.

## Conclusions

In this study, the complete genome of *L. suionicum* DSM 20241 ^T^, consisting of a circular chromosome and a circular plasmid, was obtained by whole-genome sequencing using the PacBio SMRT sequencing system and de novo assembly using the HGAP method. In addition, the metabolic pathways of organic compounds in *L. suionicum* DSM 20241 ^T^ were reconstructed to estimate its fermentative properties and metabolites. The metabolic pathways show that strain DSM 20241 ^T^ performs typical heterolactic acid fermentations to produce lactate, ethanol and carbon dioxide and contains genes encoding various PTSs, permeases, and other enzymes to metabolize various organic compounds. In addition, strain DSM 20241 ^T^ synthesizes mannitol to produce acetate instead of ethanol through heterolactic acid fermentation, and produces butter flavoring compounds. The complete genome and reconstructed metabolic pathways of *L. suionicum* DSM 20241 ^T^ provide important insights into its functional and metabolic features during fermentation.

## Additional file

Additional file 1: Table S1. List of genes of *L. suionicum* DMS 20241^T^ in KEGG metabolic pathways. (PDF 411 kb)

## Abbreviations

COG: Clusters of orthologous groups
KEGG: Kyoto Encyclopedia of Genes and Genomes
PTS: Phosphotransferase system
SMRT: Single molecule real-time
TCA: Tricarboxylic acid
