# Supplementary material for: Complete genome sequence of Leuconostoc suionicum DSM 20241T provides insights into its functional and metabolic features
Source: Stand Genomic Sci. 2017 Jul 17;12:38. doi: 10.1186/s40793-017-0256-0 (PMC5514465; doi:10.1186/s40793-017-0256-0)
Supplement: Additional file 1: Table S1. — List of genes of L. suionicum DMS 20241T in KEGG metabolic pathways. (PDF 411 kb) [file 40793_2017_256_MOESM1_ESM.pdf]

Complete genome sequence of *Leuconostoc suionicum* type strain DSM 20241 provides insights into functional and metabolic features

Byung Hee Chun[1,†], Se Hee Lee[2,†], Hye Hee Jeon[1], Dong-Woon Kim[3] and Che Ok Jeon[*,1]

[1]*Department of Life Science, Chung-Ang University, Seoul 06974, Republic of Korea*

[2]*Microbiology and Functionality Research Group, World Institute of Kimchi, Gwangju 61755, Republic of Korea*

[3]*Animal Nutrition and Physiology Team, National Institute of Animal Science, RDA, Jeollabukdo 55365, Republic of Korea*

*Author for correspondence: Che Ok Jeon (cojeon@cau.ac.kr)

[†]These authors contributed equally to this study.

Table S1. List of genes of *L. suionicum* DMS 20241^{T} in KEGG metabolic pathways.

KO number	Locus taq	Gene name
**Pentose phosphate pathway**		
K00033	Ga0151201_11564	6-Phosphogluconate dehydrogenase
K00036	Ga0151201_112041	Glucose-6-phosphate dehydrogenase
K00615	Ga0151201_111166	Transketolase
K00851	Ga0151201_11565	Tluconokinase
K00852	Ga0151201_11707	ribokinase
K00874	Ga0151201_11514	2-Dehydro-3-deoxygluconokinase
K00948	Ga0151201_11714	Ribose-phosphate pyrophosphokinase
K01621	Ga0151201_111984	Phosphoketolase
K01783	Ga0151201_111494	Ribulose-phosphate3-epimerase
K01807	Ga0151201_11145	Ribose5-phosphate isomerase A
K01810	Ga0151201_11367	Glucose-6-phosphate isomerase
K01835	Ga0151201_11619	Phosphoglucomutase
K07404	Ga0151201_11670	6-Phosphogluconolactonase
**Fructose and mannose metabolism**		
K00008	Ga0151201_11692	L-Iditol 2-dehydrogenase
K00847	Ga0151201_11289	Fructokinase
K01803	Ga0151201_11235	Triose-phosphate isomerase
K01805	Ga0151201_11118	Xylose isomerase
K01809	Ga0151201_111772	Mannose-6-phosphate isomerase
K02793	Ga0151201_111771	PTS mannose transporter subunit IIAB

K02794	Ga0151201_111251	PTS mannose-specific IIB component
K02795	Ga0151201_111250	PTS sorbose transporter subunit IIC
K02796	Ga0151201_111769	PTS mannose family transporter subunit IID
**Galactose metabolism**		
K00845	Ga0151201_11692	Glucokinase
K00849	Ga0151201_11897	Galactokinase
K00963	Ga0151201_11612	UTP-glucose-1-phosphate uridylyl transferase
K00965	Ga0151201_11898	Galactose-1-phosphate uridylyl transferase
K01182	Ga0151201_11848	Glucohydrolase
K01190	Ga0151201_111304	β-D-Galactosidase
K01193	Ga0151201_11288	β-Fructofuranosidase
K01784	Ga0151201_11282	UDP-glucose-4-epimeraseGalE
K01785	Ga0151201_111293	Galactose mutarotase
K01835	Ga0151201_11619	Phosphoglucomutase
K01854	Ga0151201_111390	UDP-galactopyranose mutase
K02744	Ga0151201_111252	PTS fructose transporter subunit IIA
K07407	Ga0151201_11896	α-galactosidase
K12308	Ga0151201_11858	β-galactosidase
**Starch and sucrose metabolism**		
K00689	Ga0151201_11873	Glycosyl hydrolase
K00690	Ga0151201_11396	Sucrose phosphorylase
K00691	Ga0151201_11907	Family 65 glycosyl hydrolase
K00694	Ga0151201_111554	Glycosyl transferase
K00845	Ga0151201_11692	Glucokinase
K00847	Ga0151201_11289	Fructokinase
K00963	Ga0151201_11612	UTP-glucose-1-phosphate uridylyl transferase
K01182	Ga0151201_11848	Glucohydrolase
K01193	Ga0151201_11288	β-fructofuranosidase
K01212	Ga0151201_11728	Sucrose-6-phosphatehydrolase
K01223	Ga0151201_11916	6-Phospho-beta-glucosidase
K01226	Ga0151201_11521	α,α-Phosphotrehalase
K01810	Ga0151201_11367	Glucose-6-phosphate isomerase
K01835	Ga0151201_11619	Phosphoglucomutase
K01838	Ga0151201_11908	β-Phosphoglucomutase
K02759	Ga0151201_11946	PTS cellbiose transporter subunit IIC
K02760	Ga0151201_11944	PTS cellobiose transporter subunit IIB
K02761	Ga0151201_11948	PTS cellobiose transporter subunit IIC
K02808	Ga0151201_11287	PTS beta-glucoside transporter subunit EIIBCA
K02809	Ga0151201_11287	PTS beta-glucoside transporter subunit EIIBCA
K02810	Ga0151201_11287	PTS beta-glucoside transporter subunit EIIBCA
K05349	Ga0151201_11890	Glycosylhydrolase

**Pyruvate metabolism**		
K00016	Ga0151201_1175	L-Lactate dehydrogenase
K00027	Ga0151201_111007	NAD-dependent malic enzyme
K00158	Ga0151201_11977	Pyruvate oxidase
K00161	Ga0151201_11753	Pyruvate dehydrogenase E1 component alpha subunit
K00162	Ga0151201_11754	α-Ketoacid dehydrogenase subunit beta
K00244	Ga0151201_11100	Flavocytochrome c
K00382	Ga0151201_11756	Dihydrolipoyl dehydrogenase
K00625	Ga0151201_111449	Phosphate acetyltransferase
K00626	Ga0151201_11809	Acetyl-CoA acetyltransferase
K00627	Ga0151201_11755	Dienelactone hydrolase
K00873	Ga0151201_11793	Pyruvate kinase
K00925	Ga0151201_11481	Acetate kinase
K01512	Ga0151201_111732	Acylphosphatase
K01595	Ga0151201_111695	Phosphoenolpyruvate carboxylase
K01649	Ga0151201_112057	2-Isopropylmalate synthase
K01961	Ga0151201_11317	Acetyl-CoA carboxylase
K01962	Ga0151201_11319	Acetyl-CoA carboxylase carboxyl transferase subunit alpha
K01963	Ga0151201_11318	Acetyl-CoA carboxylase subunit beta
K02160	Ga0151201_11315	Acetyl-CoA carboxylase biotin carboxyl carrier protein
K03778	Ga0151201_111849<br>Ga0151201_112070<br>Ga0151201_11385<br>Ga0151201_111758	D-Lactate dehydrogenase
K04072	Ga0151201_11136	Acetaldehyde dehydrogenase /alcohol dehydrogenase AdhE
